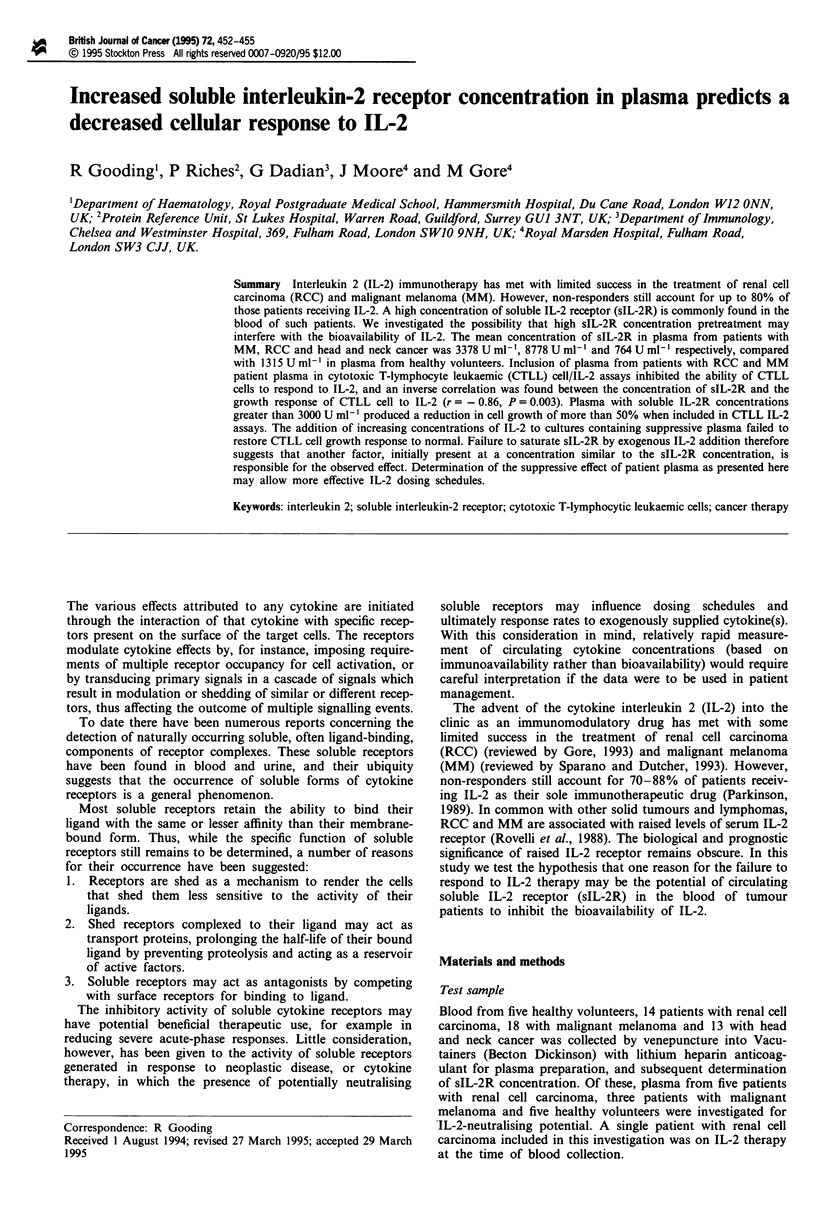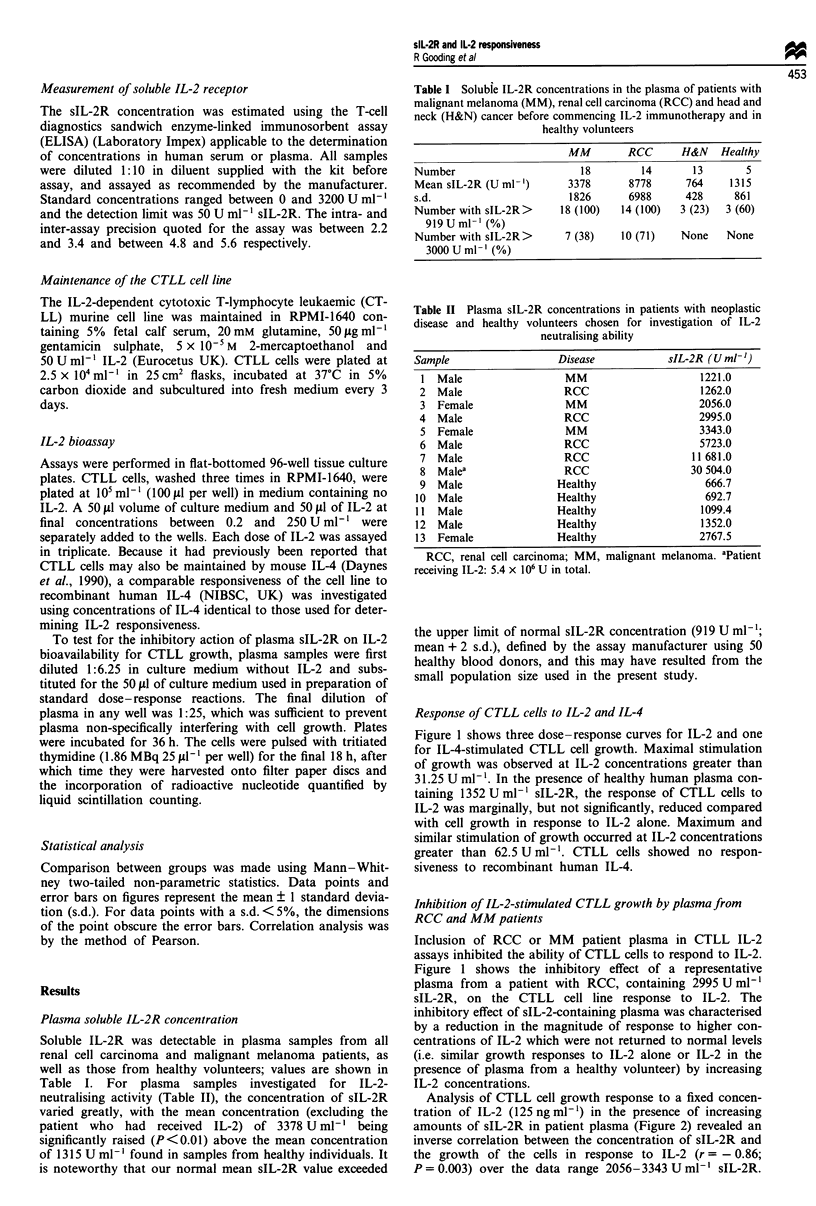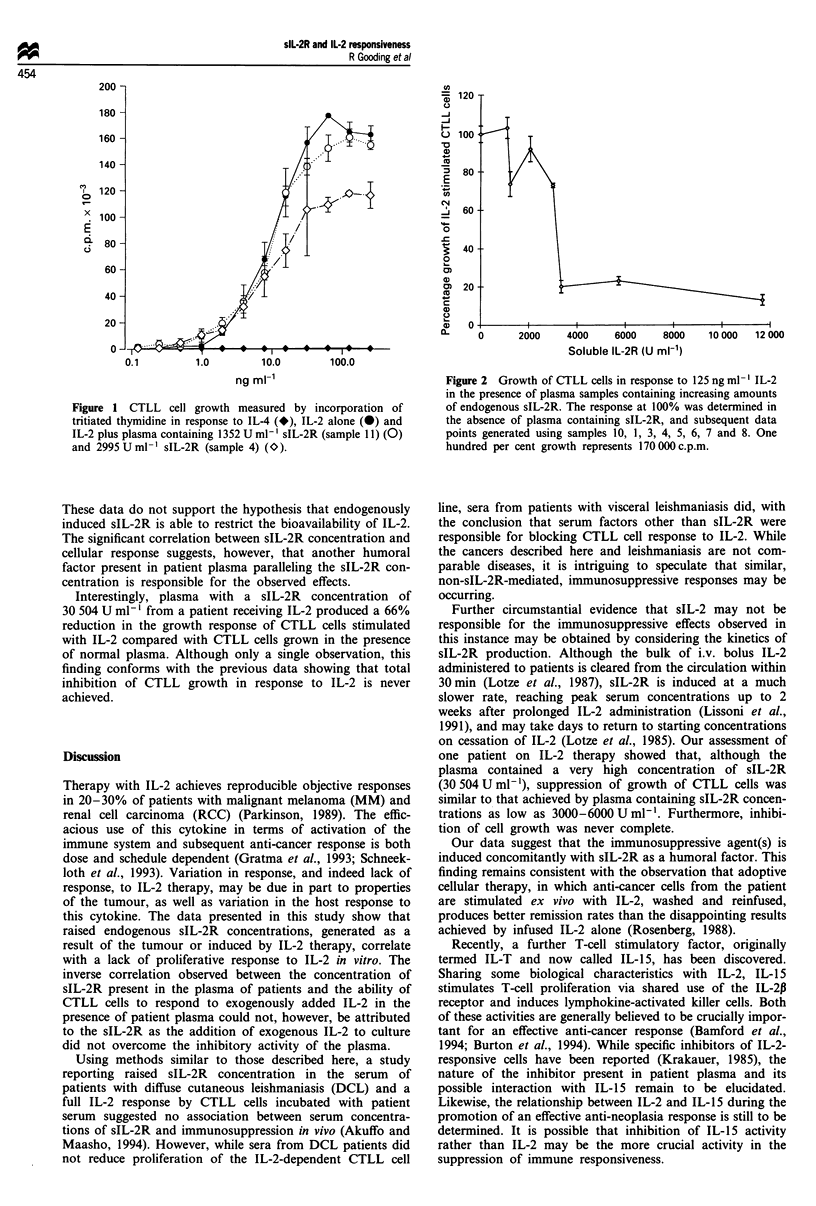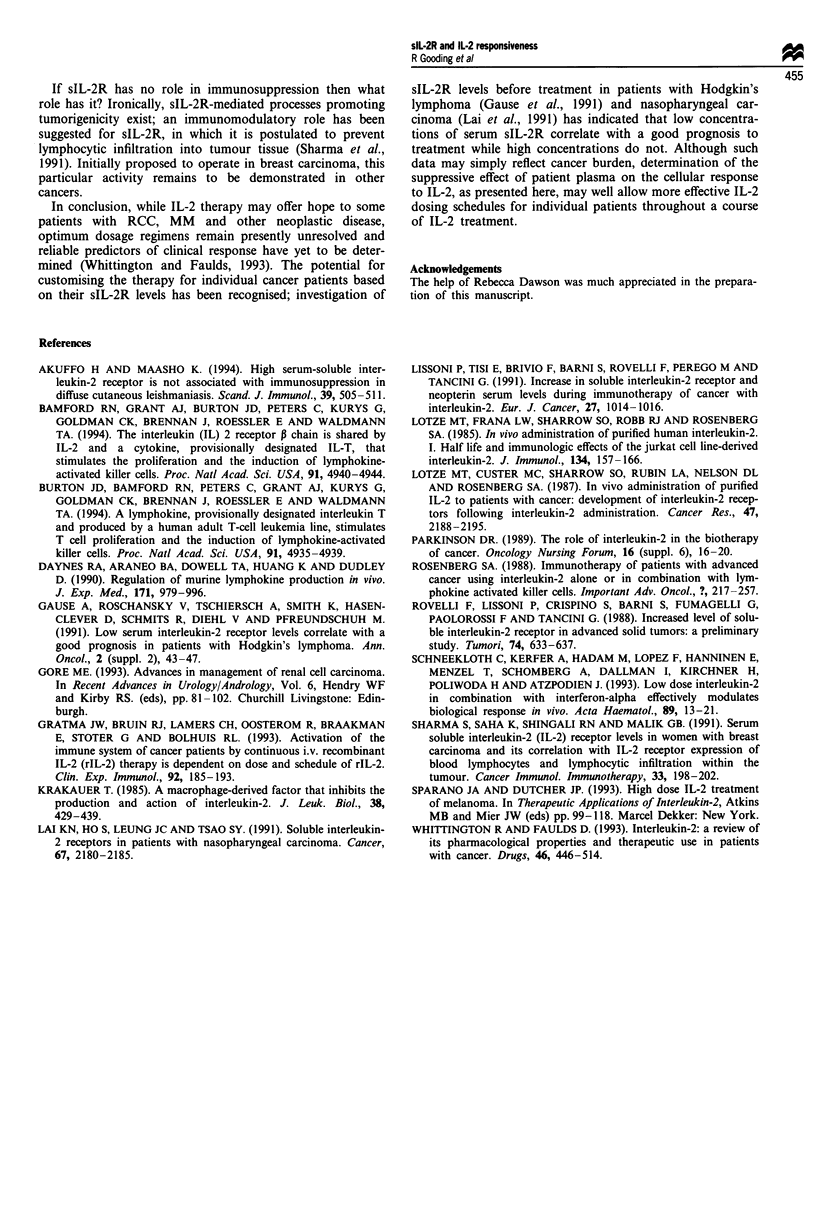# Increased soluble interleukin-2 receptor concentration in plasma predicts a decreased cellular response to IL-2.

**DOI:** 10.1038/bjc.1995.354

**Published:** 1995-08

**Authors:** R. Gooding, P. Riches, G. Dadian, J. Moore, M. Gore

**Affiliations:** Department of Haematology, Royal Postgraduate Medical School, Hammersmith Hospital, London, UK.

## Abstract

Interleukin 2 (IL-2) immunotherapy has met with limited success in the treatment of renal cell carcinoma (RCC) and malignant melanoma (MM). However, non-responders still account for up to 80% of those patients receiving IL-2. A high concentration of soluble IL-2 receptor (sIL-2R) is commonly found in the blood of such patients. We investigated the possibility that high sIL-2R concentration pretreatment may interfere with the bioavailability of IL-2. The mean concentration of sIL-2R in plasma from patients with MM, RCC and head and neck cancer was 3378 U ml-1, 8778 U ml-1 and 764 U ml-1 respectively, compared with 1315 U ml-1 in plasma from healthy volunteers. Inclusion of plasma from patients with RCC and MM patient plasma in cytotoxic T-lymphocyte leukaemic (CTLL) cell/IL-2 assays inhibited the ability of CTLL cells to respond to IL-2, and an inverse correlation was found between the concentration of sIL-2R and the growth response of CTLL cell to IL-2 (r = -0.86, P = 0.003). Plasma with soluble IL-2R concentrations greater than 3000 U ml-1 produced a reduction in cell growth of more than 50% when included in CTLL IL-2 assays. The addition of increasing concentrations of IL-2 to cultures containing suppressive plasma failed to restore CTLL cell growth response to normal. Failure to saturate sIL-2R by exogenous IL-2 addition therefore suggests that another factor, initially present at a concentration similar to the sIL-2R concentration, is responsible for the observed effect. Determination of the suppressive effect of patient plasma as presented here may allow more effective IL-2 dosing schedules.


British Journal of Cancer (1995) 72, 452-455
© 1995 Stockton Press All rights reserved 0007-0920/95 $12.00

### Increased soluble interleukin-2 receptor concentration in plasma predicts a decreased cellular response to IL-2

R Gooding[1], P Riches[2], G Dadian[3], J Moore[4] and M Gore[4]

[1]*Department of Haematology, Royal Postgraduate Medical School, Hammersmith Hospital, Du Cane Road, London W12 0NN, UK;* [2]*Protein Reference Unit, St Lukes Hospital, Warren Road, Guildford, Surrey GU1 3NT, UK;* [3]*Department of Immunology, Chelsea and Westminster Hospital, 369, Fulham Road, London SW10 9NH, UK;* [4]*Royal Marsden Hospital, Fulham Road, London SW3 CJJ, UK.*

**Summary** Interleukin 2 (IL-2) immunotherapy has met with limited success in the treatment of renal cell carcinoma (RCC) and malignant melanoma (MM). However, non-responders still account for up to 80% of those patients receiving IL-2. A high concentration of soluble IL-2 receptor (sIL-2R) is commonly found in the blood of such patients. We investigated the possibility that high sIL-2R concentration pretreatment may interfere with the bioavailability of IL-2. The mean concentration of sIL-2R in plasma from patients with MM, RCC and head and neck cancer was 3378 U ml$^{-1}$, 8778 U ml$^{-1}$ and 764 U ml$^{-1}$ respectively, compared with 1315 U ml$^{-1}$ in plasma from healthy volunteers. Inclusion of plasma from patients with RCC and MM patient plasma in cytotoxic T-lymphocyte leukaemic (CTLL) cell/IL-2 assays inhibited the ability of CTLL cells to respond to IL-2, and an inverse correlation was found between the concentration of sIL-2R and the growth response of CTLL cell to IL-2 ($r = -0.86$, $P = 0.003$). Plasma with soluble IL-2R concentrations greater than 3000 U ml$^{-1}$ produced a reduction in cell growth of more than 50% when included in CTLL IL-2 assays. The addition of increasing concentrations of IL-2 to cultures containing suppressive plasma failed to restore CTLL cell growth response to normal. Failure to saturate sIL-2R by exogenous IL-2 addition therefore suggests that another factor, initially present at a concentration similar to the sIL-2R concentration, is responsible for the observed effect. Determination of the suppressive effect of patient plasma as presented here may allow more effective IL-2 dosing schedules.

**Keywords**: interleukin 2; soluble interleukin-2 receptor; cytotoxic T-lymphocytic leukaemic cells; cancer therapy

The various effects attributed to any cytokine are initiated through the interaction of that cytokine with specific receptors present on the surface of the target cells. The receptors modulate cytokine effects by, for instance, imposing requirements of multiple receptor occupancy for cell activation, or by transducing primary signals in a cascade of signals which result in modulation or shedding of similar or different receptors, thus affecting the outcome of multiple signalling events.

To date there have been numerous reports concerning the detection of naturally occurring soluble, often ligand-binding, components of receptor complexes. These soluble receptors have been found in blood and urine, and their ubiquity suggests that the occurrence of soluble forms of cytokine receptors is a general phenomenon.

Most soluble receptors retain the ability to bind their ligand with the same or lesser affinity than their membrane-bound form. Thus, while the specific function of soluble receptors still remains to be determined, a number of reasons for their occurrence have been suggested:

1. Receptors are shed as a mechanism to render the cells that shed them less sensitive to the activity of their ligands.
2. Shed receptors complexed to their ligand may act as transport proteins, prolonging the half-life of their bound ligand by preventing proteolysis and acting as a reservoir of active factors.
3. Soluble receptors may act as antagonists by competing with surface receptors for binding to ligand.

The inhibitory activity of soluble cytokine receptors may have potential beneficial therapeutic use, for example in reducing severe acute-phase responses. Little consideration, however, has been given to the activity of soluble receptors generated in response to neoplastic disease, or cytokine therapy, in which the presence of potentially neutralising soluble receptors may influence dosing schedules and ultimately response rates to exogenously supplied cytokine(s). With this consideration in mind, relatively rapid measurement of circulating cytokine concentrations (based on immunoavailability rather than bioavailability) would require careful interpretation if the data were to be used in patient management.

The advent of the cytokine interleukin 2 (IL-2) into the clinic as an immunomodulatory drug has met with some limited success in the treatment of renal cell carcinoma (RCC) (reviewed by Gore, 1993) and malignant melanoma (MM) (reviewed by Sparano and Dutcher, 1993). However, non-responders still account for 70-88% of patients receiving IL-2 as their sole immunotherapeutic drug (Parkinson, 1989). In common with other solid tumours and lymphomas, RCC and MM are associated with raised levels of serum IL-2 receptor (Rovelli *et al.*, 1988). The biological and prognostic significance of raised IL-2 receptor remains obscure. In this study we test the hypothesis that one reason for the failure to respond to IL-2 therapy may be the potential of circulating soluble IL-2 receptor (sIL-2R) in the blood of tumour patients to inhibit the bioavailability of IL-2.

### Materials and methods

### *Test sample*

Blood from five healthy volunteers, 14 patients with renal cell carcinoma, 18 with malignant melanoma and 13 with head and neck cancer was collected by venepuncture into Vacutainers (Becton Dickinson) with lithium heparin anticoagulant for plasma preparation, and subsequent determination of sIL-2R concentration. Of these, plasma from five patients with renal cell carcinoma, three patients with malignant melanoma and five healthy volunteers were investigated for IL-2-neutralising potential. A single patient with renal cell carcinoma included in this investigation was on IL-2 therapy at the time of blood collection.

Correspondence: R Gooding
Received 1 August 1994; revised 27 March 1995; accepted 29 March 1995

*Measurement of soluble IL-2 receptor*

The sIL-2R concentration was estimated using the T-cell diagnostics sandwich enzyme-linked immunosorbent assay (ELISA) (Laboratory Impex) applicable to the determination of concentrations in human serum or plasma. All samples were diluted 1:10 in diluent supplied with the kit before assay, and assayed as recommended by the manufacturer. Standard concentrations ranged between 0 and 3200 U $ml^{-1}$ and the detection limit was 50 U $ml^{-1}$ sIL-2R. The intra- and inter-assay precision quoted for the assay was between 2.2 and 3.4 and between 4.8 and 5.6 respectively.

*Maintenance of the CTLL cell line*

The IL-2-dependent cytotoxic T-lymphocyte leukaemic (CTLL) murine cell line was maintained in RPMI-1640 containing 5% fetal calf serum, 20 mM glutamine, 50 μg $ml^{-1}$ gentamicin sulphate, $5 \times 10^{-5}$ M 2-mercaptoethanol and 50 U $ml^{-1}$ IL-2 (Eurocetus UK). CTLL cells were plated at $2.5 \times 10^4$ $ml^{-1}$ in 25 $cm^2$ flasks, incubated at 37°C in 5% carbon dioxide and subcultured into fresh medium every 3 days.

*IL-2 bioassay*

Assays were performed in flat-bottomed 96-well tissue culture plates. CTLL cells, washed three times in RPMI-1640, were plated at $10^5$ $ml^{-1}$ (100 μl per well) in medium containing no IL-2. A 50 μl volume of culture medium and 50 μl of IL-2 at final concentrations between 0.2 and 250 U $ml^{-1}$ were separately added to the wells. Each dose of IL-2 was assayed in triplicate. Because it had previously been reported that CTLL cells may also be maintained by mouse IL-4 (Daynes *et al.*, 1990), a comparable responsiveness of the cell line to recombinant human IL-4 (NIBSC, UK) was investigated using concentrations of IL-4 identical to those used for determining IL-2 responsiveness.

To test for the inhibitory action of plasma sIL-2R on IL-2 bioavailability for CTLL growth, plasma samples were first diluted 1:6.25 in culture medium without IL-2 and substituted for the 50 μl of culture medium used in preparation of standard dose–response reactions. The final dilution of plasma in any well was 1:25, which was sufficient to prevent plasma non-specifically interfering with cell growth. Plates were incubated for 36 h. The cells were pulsed with tritiated thymidine (1.86 MBq 25 $\mu l^{-1}$ per well) for the final 18 h, after which time they were harvested onto filter paper discs and the incorporation of radioactive nucleotide quantified by liquid scintillation counting.

*Statistical analysis*

Comparison between groups was made using Mann–Whitney two-tailed non-parametric statistics. Data points and error bars on figures represent the mean ± 1 standard deviation (s.d.). For data points with a s.d. < 5%, the dimensions of the point obscure the error bars. Correlation analysis was by the method of Pearson.

### Results

*Plasma soluble IL-2R concentration*

Soluble IL-2R was detectable in plasma samples from all renal cell carcinoma and malignant melanoma patients, as well as those from healthy volunteers; values are shown in Table I. For plasma samples investigated for IL-2-neutralising activity (Table II), the concentration of sIL-2R varied greatly, with the mean concentration (excluding the patient who had received IL-2) of 3378 U $ml^{-1}$ being significantly raised ($P < 0.01$) above the mean concentration of 1315 U $ml^{-1}$ found in samples from healthy individuals. It is noteworthy that our normal mean sIL-2R value exceeded the upper limit of normal sIL-2R concentration (919 U $ml^{-1}$; mean + 2 s.d.), defined by the assay manufacturer using 50 healthy blood donors, and this may have resulted from the small population size used in the present study.

**Table I** Soluble IL-2R concentrations in the plasma of patients with malignant melanoma (MM), renal cell carcinoma (RCC) and head and neck (H&N) cancer before commencing IL-2 immunotherapy and in healthy volunteers

| | *MM* | *RCC* | *H&N* | *Healthy* |
|---|---|---|---|---|
| Number | 18 | 14 | 13 | 5 |
| Mean sIL-2R (U $ml^{-1}$) | 3378 | 8778 | 764 | 1315 |
| s.d. | 1826 | 6988 | 428 | 861 |
| Number with sIL-2R > 919 U $ml^{-1}$ (%) | 18 (100) | 14 (100) | 3 (23) | 3 (60) |
| Number with sIL-2R > 3000 U $ml^{-1}$ (%) | 7 (38) | 10 (71) | None | None |

**Table II** Plasma sIL-2R concentrations in patients with neoplastic disease and healthy volunteers chosen for investigation of IL-2 neutralising ability

| *Sample* | *Disease* | *sIL-2R (U $ml^{-1}$)* |
|---|---|---|
| 1 Male | MM | 1221.0 |
| 2 Male | RCC | 1262.0 |
| 3 Female | MM | 2056.0 |
| 4 Male | RCC | 2995.0 |
| 5 Female | MM | 3343.0 |
| 6 Male | RCC | 5723.0 |
| 7 Male | RCC | 11 681.0 |
| 8 Male[a] | RCC | 30 504.0 |
| 9 Male | Healthy | 666.7 |
| 10 Male | Healthy | 692.7 |
| 11 Male | Healthy | 1099.4 |
| 12 Male | Healthy | 1352.0 |
| 13 Female | Healthy | 2767.5 |

RCC, renal cell carcinoma; MM, malignant melanoma. [a]Patient receiving IL-2: $5.4 \times 10^6$ U in total.

*Response of CTLL cells to IL-2 and IL-4*

Figure 1 shows three dose–response curves for IL-2 and one for IL-4-stimulated CTLL cell growth. Maximal stimulation of growth was observed at IL-2 concentrations greater than 31.25 U $ml^{-1}$. In the presence of healthy human plasma containing 1352 U $ml^{-1}$ sIL-2R, the response of CTLL cells to IL-2 was marginally, but not significantly, reduced compared with cell growth in response to IL-2 alone. Maximum and similar stimulation of growth occurred at IL-2 concentrations greater than 62.5 U $ml^{-1}$. CTLL cells showed no responsiveness to recombinant human IL-4.

*Inhibition of IL-2-stimulated CTLL growth by plasma from RCC and MM patients*

Inclusion of RCC or MM patient plasma in CTLL IL-2 assays inhibited the ability of CTLL cells to respond to IL-2. Figure 1 shows the inhibitory effect of a representative plasma from a patient with RCC, containing 2995 U $ml^{-1}$ sIL-2R, on the CTLL cell line response to IL-2. The inhibitory effect of sIL-2-containing plasma was characterised by a reduction in the magnitude of response to higher concentrations of IL-2 which were not returned to normal levels (i.e. similar growth responses to IL-2 alone or IL-2 in the presence of plasma from a healthy volunteer) by increasing IL-2 concentrations.

Analysis of CTLL cell growth response to a fixed concentration of IL-2 (125 ng $ml^{-1}$) in the presence of increasing amounts of sIL-2R in patient plasma (Figure 2) revealed an inverse correlation between the concentration of sIL-2R and the growth of the cells in response to IL-2 ($r = -0.86$; $P = 0.003$) over the data range 2056–3343 U $ml^{-1}$ sIL-2R.

Figure 1 CTLL cell growth measured by incorporation of tritiated thymidine in response to IL-4 (◆), IL-2 alone (●) and IL-2 plus plasma containing 1352 U ml$^{-1}$ sIL-2R (sample 11) (○) and 2995 U ml$^{-1}$ sIL-2R (sample 4) (◇).

Figure 2 Growth of CTLL cells in response to 125 ng ml$^{-1}$ IL-2 in the presence of plasma samples containing increasing amounts of endogenous sIL-2R. The response at 100% was determined in the absence of plasma containing sIL-2R, and subsequent data points generated using samples 10, 1, 3, 4, 5, 6, 7 and 8. One hundred per cent growth represents 170 000 c.p.m.

These data do not support the hypothesis that endogenously induced sIL-2R is able to restrict the bioavailability of IL-2. The significant correlation between sIL-2R concentration and cellular response suggests, however, that another humoral factor present in patient plasma paralleling the sIL-2R concentration is responsible for the observed effects.

Interestingly, plasma with a sIL-2R concentration of 30 504 U ml$^{-1}$ from a patient receiving IL-2 produced a 66% reduction in the growth response of CTLL cells stimulated with IL-2 compared with CTLL cells grown in the presence of normal plasma. Although only a single observation, this finding conforms with the previous data showing that total inhibition of CTLL growth in response to IL-2 is never achieved.

### Discussion

Therapy with IL-2 achieves reproducible objective responses in 20–30% of patients with malignant melanoma (MM) and renal cell carcinoma (RCC) (Parkinson, 1989). The efficacious use of this cytokine in terms of activation of the immune system and subsequent anti-cancer response is both dose and schedule dependent (Gratma *et al.*, 1993; Schneekloth *et al.*, 1993). Variation in response, and indeed lack of response, to IL-2 therapy, may be due in part to properties of the tumour, as well as variation in the host response to this cytokine. The data presented in this study show that raised endogenous sIL-2R concentrations, generated as a result of the tumour or induced by IL-2 therapy, correlate with a lack of proliferative response to IL-2 *in vitro*. The inverse correlation observed between the concentration of sIL-2R present in the plasma of patients and the ability of CTLL cells to respond to exogenously added IL-2 in the presence of patient plasma could not, however, be attributed to the sIL-2R as the addition of exogenous IL-2 to culture did not overcome the inhibitory activity of the plasma.

Using methods similar to those described here, a study reporting raised sIL-2R concentration in the serum of patients with diffuse cutaneous leishmaniasis (DCL) and a full IL-2 response by CTLL cells incubated with patient serum suggested no association between serum concentrations of sIL-2R and immunosuppression *in vivo* (Akuffo and Maasho, 1994). However, while sera from DCL patients did not reduce proliferation of the IL-2-dependent CTLL cell line, sera from patients with visceral leishmaniasis did, with the conclusion that serum factors other than sIL-2R were responsible for blocking CTLL cell response to IL-2. While the cancers described here and leishmaniasis are not comparable diseases, it is intriguing to speculate that similar, non-sIL-2R-mediated, immunosuppressive responses may be occurring.

Further circumstantial evidence that sIL-2 may not be responsible for the immunosuppressive effects observed in this instance may be obtained by considering the kinetics of sIL-2R production. Although the bulk of i.v. bolus IL-2 administered to patients is cleared from the circulation within 30 min (Lotze *et al.*, 1987), sIL-2R is induced at a much slower rate, reaching peak serum concentrations up to 2 weeks after prolonged IL-2 administration (Lissoni *et al.*, 1991), and may take days to return to starting concentrations on cessation of IL-2 (Lotze *et al.*, 1985). Our assessment of one patient on IL-2 therapy showed that, although the plasma contained a very high concentration of sIL-2R (30 504 U ml$^{-1}$), suppression of growth of CTLL cells was similar to that achieved by plasma containing sIL-2R concentrations as low as 3000–6000 U ml$^{-1}$. Furthermore, inhibition of cell growth was never complete.

Our data suggest that the immunosuppressive agent(s) is induced concomitantly with sIL-2R as a humoral factor. This finding remains consistent with the observation that adoptive cellular therapy, in which anti-cancer cells from the patient are stimulated *ex vivo* with IL-2, washed and reinfused, produces better remission rates than the disappointing results achieved by infused IL-2 alone (Rosenberg, 1988).

Recently, a further T-cell stimulatory factor, originally termed IL-T and now called IL-15, has been discovered. Sharing some biological characteristics with IL-2, IL-15 stimulates T-cell proliferation via shared use of the IL-2$\beta$ receptor and induces lymphokine-activated killer cells. Both of these activities are generally believed to be crucially important for an effective anti-cancer response (Bamford *et al.*, 1994; Burton *et al.*, 1994). While specific inhibitors of IL-2-responsive cells have been reported (Krakauer, 1985), the nature of the inhibitor present in patient plasma and its possible interaction with IL-15 remain to be elucidated. Likewise, the relationship between IL-2 and IL-15 during the promotion of an effective anti-neoplasia response is still to be determined. It is possible that inhibition of IL-15 activity rather than IL-2 may be the more crucial activity in the suppression of immune responsiveness.

If sIL-2R has no role in immunosuppression then what role has it? Ironically, sIL-2R-mediated processes promoting tumorigenicity exist; an immunomodulatory role has been suggested for sIL-2R, in which it is postulated to prevent lymphocytic infiltration into tumour tissue (Sharma *et al.*, 1991). Initially proposed to operate in breast carcinoma, this particular activity remains to be demonstrated in other cancers.

In conclusion, while IL-2 therapy may offer hope to some patients with RCC, MM and other neoplastic disease, optimum dosage regimens remain presently unresolved and reliable predictors of clinical response have yet to be determined (Whittington and Faulds, 1993). The potential for customising the therapy for individual cancer patients based on their sIL-2R levels has been recognised; investigation of sIL-2R levels before treatment in patients with Hodgkin's lymphoma (Gause *et al.*, 1991) and nasopharyngeal carcinoma (Lai *et al.*, 1991) has indicated that low concentrations of serum sIL-2R correlate with a good prognosis to treatment while high concentrations do not. Although such data may simply reflect cancer burden, determination of the suppressive effect of patient plasma on the cellular response to IL-2, as presented here, may well allow more effective IL-2 dosing schedules for individual patients throughout a course of IL-2 treatment.

**Acknowledgements**
The help of Rebecca Dawson was much appreciated in the preparation of this manuscript.

### References

AKUFFO H AND MAASHO K. (1994). High serum-soluble interleukin-2 receptor is not associated with immunosuppression in diffuse cutaneous leishmaniasis. *Scand. J. Immunol.*, **39**, 505–511.

BAMFORD RN, GRANT AJ, BURTON JD, PETERS C, KURYS G, GOLDMAN CK, BRENNAN J, ROESSLER E AND WALDMANN TA. (1994). The interleukin (IL) 2 receptor β chain is shared by IL-2 and a cytokine, provisionally designated IL-T, that stimulates the proliferation and the induction of lymphokine-activated killer cells. *Proc. Natl Acad. Sci. USA*, **91**, 4940–4944.

BURTON JD, BAMFORD RN, PETERS C, GRANT AJ, KURYS G, GOLDMAN CK, BRENNAN J, ROESSLER E AND WALDMANN TA. (1994). A lymphokine, provisionally designated interleukin T and produced by a human adult T-cell leukemia line, stimulates T cell proliferation and the induction of lymphokine-activated killer cells. *Proc. Natl Acad. Sci. USA*, **91**, 4935–4939.

DAYNES RA, ARANEO BA, DOWELL TA, HUANG K AND DUDLEY D. (1990). Regulation of murine lymphokine production *in vivo*. *J. Exp. Med.*, **171**, 979–996.

GAUSE A, ROSCHANSKY V, TSCHIERSCH A, SMITH K, HASENCLEVER D, SCHMITS R, DIEHL V AND PFREUNDSCHUH M. (1991). Low serum interleukin-2 receptor levels correlate with a good prognosis in patients with Hodgkin's lymphoma. *Ann. Oncol.*, **2** (suppl. 2), 43–47.

GORE ME. (1993). Advances in management of renal cell carcinoma. In *Recent Advances in Urology/Andrology*, Vol. 6, Hendry WF and Kirby RS. (eds), pp. 81–102. Churchill Livingstone: Edinburgh.

GRATMA JW, BRUIN RJ, LAMERS CH, OOSTEROM R, BRAAKMAN E, STOTER G AND BOLHUIS RL. (1993). Activation of the immune system of cancer patients by continuous i.v. recombinant IL-2 (rIL-2) therapy is dependent on dose and schedule of rIL-2. *Clin. Exp. Immunol.*, **92**, 185–193.

KRAKAUER T. (1985). A macrophage-derived factor that inhibits the production and action of interleukin-2. *J. Leuk. Biol.*, **38**, 429–439.

LAI KN, HO S, LEUNG JC AND TSAO SY. (1991). Soluble interleukin-2 receptors in patients with nasopharyngeal carcinoma. *Cancer*, **67**, 2180–2185.

LISSONI P, TISI E, BRIVIO F, BARNI S, ROVELLI F, PEREGO M AND TANCINI G. (1991). Increase in soluble interleukin-2 receptor and neopterin serum levels during immunotherapy of cancer with interleukin-2. *Eur. J. Cancer*, **27**, 1014–1016.

LOTZE MT, FRANA LW, SHARROW SO, ROBB RJ AND ROSENBERG SA. (1985). *In vivo* administration of purified human interleukin-2. I. Half life and immunologic effects of the jurkat cell line-derived interleukin-2. *J. Immunol.*, **134**, 157–166.

LOTZE MT, CUSTER MC, SHARROW SO, RUBIN LA, NELSON DL AND ROSENBERG SA. (1987). In vivo administration of purified IL-2 to patients with cancer: development of interleukin-2 receptors following interleukin-2 administration. *Cancer Res.*, **47**, 2188–2195.

PARKINSON DR. (1989). The role of interleukin-2 in the biotherapy of cancer. *Oncology Nursing Forum*, **16** (suppl. 6), 16–20.

ROSENBERG SA. (1988). Immunotherapy of patients with advanced cancer using interleukin-2 alone or in combination with lymphokine activated killer cells. *Important Adv. Oncol.*, **?**, 217–257.

ROVELLI F, LISSONI P, CRISPINO S, BARNI S, FUMAGELLI G, PAOLOROSSI F AND TANCINI G. (1988). Increased level of soluble interleukin-2 receptor in advanced solid tumors: a preliminary study. *Tumori*, **74**, 633–637.

SCHNEEKLOTH C, KERFER A, HADAM M, LOPEZ F, HANNINEN E, MENZEL T, SCHOMBERG A, DALLMAN I, KIRCHNER H, POLIWODA H AND ATZPODIEN J. (1993). Low dose interleukin-2 in combination with interferon-alpha effectively modulates biological response *in vivo*. *Acta Haematol.*, **89**, 13–21.

SHARMA S, SAHA K, SHINGALI RN AND MALIK GB. (1991). Serum soluble interleukin-2 (IL-2) receptor levels in women with breast carcinoma and its correlation with IL-2 receptor expression of blood lymphocytes and lymphocytic infiltration within the tumour. *Cancer Immunol. Immunotherapy*, **33**, 198–202.

SPARANO JA AND DUTCHER JP. (1993). High dose IL-2 treatment of melanoma. In *Therapeutic Applications of Interleukin-2*, Atkins MB and Mier JW (eds) pp. 99–118. Marcel Dekker: New York.

WHITTINGTON R AND FAULDS D. (1993). Interleukin-2: a review of its pharmacological properties and therapeutic use in patients with cancer. *Drugs*, **46**, 446–514.